# *catena*-Poly[[[di­aqua­cobalt(II)]-bis­{μ-2-[3-(4-carboxyl­atophen­yl)pyridin-1-ium-1-yl]acetato}] dihydrate]

**DOI:** 10.1107/S1600536813008933

**Published:** 2013-04-10

**Authors:** Wei Gao, Xiu-Mei Zhang

**Affiliations:** a College of Chemistry and Materials Science, Huaibei Normal University, 100 Dongshan Road, Huaibei, Anhui 235000, People’s Republic of China

## Abstract

In the title polymeric coordination compound, {[Co(C_14_H_10_NO_4_)_2_(H_2_O)_2_]·2H_2_O}_*n*_, the Co^II^ ion resides on an inversion center and exhibits a distorted o­cta­hedral coordination geometry defined by four O atoms from two pairs of equivalent monodentate carboxyl­ate groups from 2-[3-(4-carboxyl­atophen­yl)pyridin-1-ium-1-yl]acetate ligands and by two O atoms from two equivalent coordinating water mol­ecules. The zwitterionic di­carboxyl­ate ligands serve as bridges with two monodentate carboxyl­ate and the metal ions are linked by double bridges, forming polymeric chains running along [01-1]. The chains are further stabilized and associated into layers parallel to (011) through intra- and inter­chain hydrogen bonding and π–π stacking inter­actions [inter­planar and centroid–centroid distances of 3.658 (3) Å and 3.653 (2) Å, respectively].

## Related literature

For general background to zwitterionic ligands that contain more carboxyl­ate groups than positive groups and hence have reduced negative charge, see: Zhang *et al.* (2010 ▶); Wang *et al.* (2009 ▶). For the synthesis of the ligand, see: Loeb *et al.* (2006 ▶).
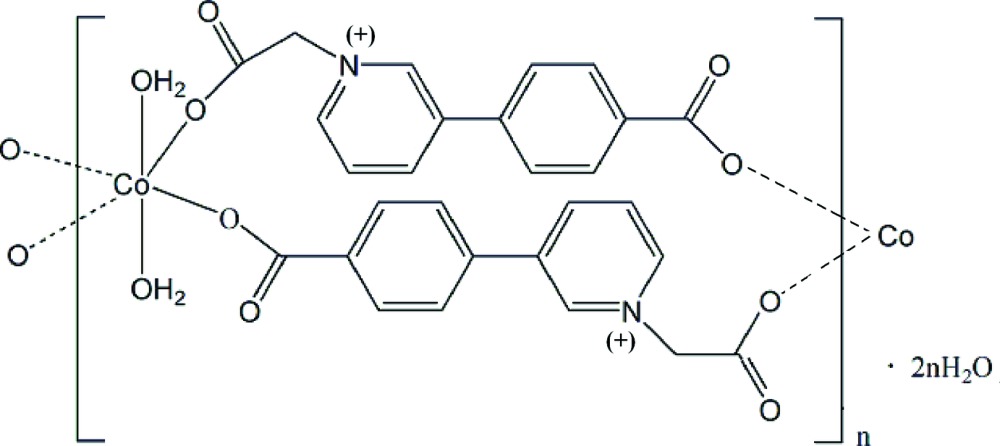


## Experimental

### 

#### Crystal data

[Co(C_14_H_10_NO_4_)_2_(H_2_O)_2_]·2H_2_O
*M* *_r_* = 643.45
Triclinic, 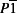

*a* = 7.5943 (3) Å
*b* = 7.9123 (3) Å
*c* = 10.7673 (4) Å
α = 88.769 (1)°
β = 81.681 (1)°
γ = 83.920 (1)°
*V* = 636.57 (4) Å^3^
*Z* = 1
Mo *K*α radiation
μ = 0.75 mm^−1^
*T* = 296 K
0.10 × 0.08 × 0.06 mm

#### Data collection

Bruker SMART CCD area-detector diffractometer
Absorption correction: multi-scan (*SADABS*; Bruker, 2008 ▶) *T*_min_ = 0.929, *T*_max_ = 0.956
7933 measured reflections
2477 independent reflections
2449 reflections with *I* > 2σ(*I*)
*R*_int_ = 0.015

#### Refinement

*R*[*F* ^2^ > 2σ(*F* ^2^)] = 0.030
*wR*(*F* ^2^) = 0.091
*S* = 1.05
2477 reflections
208 parameters
3 restraints
H atoms treated by a mixture of independent and constrained refinement
Δρ_max_ = 0.42 e Å^−3^
Δρ_min_ = −0.43 e Å^−3^

### 

Data collection: *SMART* (Bruker, 2007 ▶); cell refinement: *SAINT* (Bruker, 2007 ▶); data reduction: *SAINT*; program(s) used to solve structure: *SHELXTL* (Sheldrick, 2008 ▶); program(s) used to refine structure: *SHELXTL*; molecular graphics: *SHELXTL*; software used to prepare material for publication: *SHELXTL*.

## Supplementary Material

Crystal structure: contains datablock(s) I, global. DOI: 10.1107/S1600536813008933/bg2502sup1.cif

Structure factors: contains datablock(s) I. DOI: 10.1107/S1600536813008933/bg2502Isup2.hkl

Additional supplementary materials:  crystallographic information; 3D view; checkCIF report

## Figures and Tables

**Table 1:** Hydrogen-bond geometry (Å, °)

*D*—H⋯*A*	*D*—H	H⋯*A*	*D*⋯*A*	*D*—H⋯*A*
O5—H5*C*⋯O2^i^	0.95 (3)	1.91 (3)	2.835 (2)	164 (3)
O5—H5*B*⋯O3^ii^	0.85 (3)	1.80 (3)	2.617 (2)	162 (3)
O6—H6*A*⋯O4^iii^	0.93 (2)	2.13 (2)	3.003 (2)	156 (3)
O6—H6*B*⋯O5^iv^	0.92 (2)	1.96 (2)	2.880 (2)	173 (4)

Symmetry codes: (i) 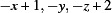; (ii) 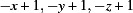; (iii) 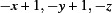; (iv) 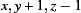.

**Fig. 1.**
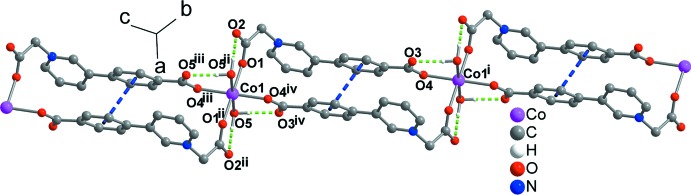
The one-dimensional coordination chain with the π-π stacking and hydrogen bonds intrachain the chains. The intrachain hydrogen bonds and π-π stacking are shown as dot lines. [Symmetry codes: (i)-x + 1, -y + 1, -z + 1 (iii) -x + 1, -y, -z + 2 (iv)x, y + 1, z - 1]

**Fig. 2.**
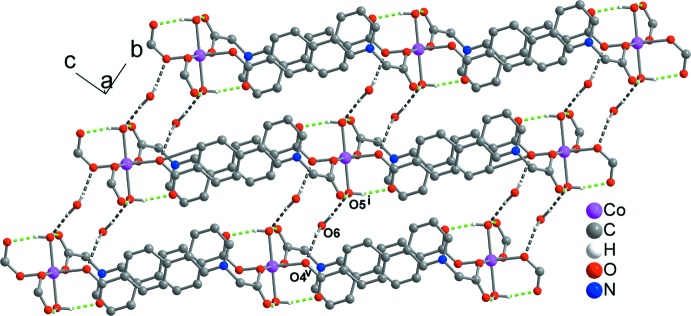
The two-dimensional layer assembled through hydrogen bonding interactions. [Symmetry codes: (i)-x + 1, -y + 1, -z + 1 (v)-x + 1, -y + 1, -z]

## supplementary crystallographic information

### Comment

The zwitterionic ligands that contain more carboxylate groups than positive groups and hence have reduced negative charge have received little attention in crystal engineering and coordination chemistry (Zhang *et al.* (2010); Wang *et al.*. (2009)). The charge on the carboxylate ligand will certainly influence the coordination and supramolecular structures.

In this paper, we report the coordination and hydrogen-bond structure of the title Co^II^ complex (I) derived from the zwitterionic ligand 3-carboxymethylpyridinium-4-benzoate (*L*).

The asymmetric unit of I contains a Co^II^ ion on a centre of symmetry, one *L* ligand, one coordinated water molecule, and one lattice water molecule. Each Co atom resides in a distorted octahedral coordination geometry completed by four carboxylate O atoms from four *L* ligands and two O atoms from two coordinated water molecules. The Co—O distances lie in the range of 2.1031 (12)–2.1392 (14) Å. The *L* ligand binds two Co atoms through two monodentate carboxylate groups. Consequently, adjacent Co^II^ centers are connected by a pair of zwitterionic ligands to give one-dimensional chains running along [011] (Fig.1). These coordination chains are further reinforced by the π-π interaction between the centrosymmetry-related phenylene groups (the interplanar and center-to-center distances are 3.658 (3) Å and 3.653 (2) Å respectively). Neighboring chains are associated *via* O—H···O hydrogen bonds mediated by lattice water molecules, which donate one hydrogen atom to a coordinated oxygen carboxylate from one chain and to a coordinated water molecule from another chain. Consequently, the chains are linked into layers (Fig. 2). The hydrogen bonding parameters are listed in Table 1.

### Experimental

The ligand was synthesized from 4-(3-pyridyl)benzoic acid and ethyl bromoacetate according to the procedure for similar compounds (Loeb *et al.*, 2006). A mixture of the ligand (0.024 g, 0.10 mmol) and CoCl_2_.6H_2_O (0.010 g, 0.050 mmol) was thoroughly mixed in H_2_O (2 ml) and CH_3_OH (2 ml) in a Teflon-lined stainless steel vessel (25 ml), heated at 70°C for two days under autogenous pressure, and then cooled to room temperature. Red block crystals were harvested.

### Refinement

All the hydrogen atoms attached to carbon atoms were placed in calculated positions and refined using the riding model, and the water hydrogen atoms were located from the difference maps.

### Crystal data

**Table:**

[Co(C_14_H_10_NO_4_)_2_(H_2_O)_2_]·2H_2_O	*V* = 636.57 (4) Å^3^
*M**_r_* = 643.45	*Z* = 1
Triclinic, *P*1	*F*(000) = 333
Hall symbol: -P 1	*D*_x_ = 1.679 Mg m^−^^3^
*a* = 7.5943 (3) Å	Mo *K*α radiation, λ = 0.71073 Å
*b* = 7.9123 (3) Å	µ = 0.75 mm^−^^1^
*c* = 10.7673 (4) Å	*T* = 296 K
α = 88.769 (1)°	Block, red
β = 81.681 (1)°	0.10 × 0.08 × 0.06 mm
γ = 83.920 (1)°	

### Data collection

**Table:**

Bruker SMART CCD area-detector diffractometer	2477 independent reflections
Radiation source: fine-focus sealed tube	2449 reflections with *I* > 2σ(*I*)
Graphite monochromator	*R*_int_ = 0.015
phi and ω scans	θ_max_ = 26.0°, θ_min_ = 1.9°
Absorption correction: multi-scan (*SADABS*; Bruker, 2008)	*h* = −9→9
*T*_min_ = 0.929, *T*_max_ = 0.956	*k* = −9→9
7933 measured reflections	*l* = −10→13

### Refinement

**Table:**

Refinement on *F*^2^	Primary atom site location: structure-invariant direct methods
Least-squares matrix: full	Secondary atom site location: difference Fourier map
*R*[*F*^2^ > 2σ(*F*^2^)] = 0.030	Hydrogen site location: inferred from neighbouring sites
*wR*(*F*^2^) = 0.091	H atoms treated by a mixture of independent and constrained refinement
*S* = 1.05	*w* = 1/[σ^2^(*F*_o_^2^) + (0.0573*P*)^2^ + 0.4307*P*] where *P* = (*F*_o_^2^ + 2*F*_c_^2^)/3
2477 reflections	(Δ/σ)_max_ < 0.001
208 parameters	Δρ_max_ = 0.42 e Å^−^^3^
3 restraints	Δρ_min_ = −0.43 e Å^−^^3^

### Special details

**Table:**

Geometry. All e.s.d.'s (except the e.s.d. in the dihedral angle between two l.s. planes) are estimated using the full covariance matrix. The cell e.s.d.'s are taken into account individually in the estimation of e.s.d.'s in distances, angles and torsion angles; correlations between e.s.d.'s in cell parameters are only used when they are defined by crystal symmetry. An approximate (isotropic) treatment of cell e.s.d.'s is used for estimating e.s.d.'s involving l.s. planes.
Refinement. Refinement of *F*^2^ against ALL reflections. The weighted *R*-factor *wR* and goodness of fit *S* are based on *F*^2^, conventional *R*-factors *R* are based on *F*, with *F* set to zero for negative *F*^2^. The threshold expression of *F*^2^ > σ(*F*^2^) is used only for calculating *R*-factors(gt) *etc*. and is not relevant to the choice of reflections for refinement. *R*-factors based on *F*^2^ are statistically about twice as large as those based on *F*, and *R*- factors based on ALL data will be even larger.

### Fractional atomic coordinates and isotropic or equivalent isotropic displacement parameters (Å^2^)

**Table:**

	*x*	*y*	*z*	*U*_iso_*/*U*_eq_	
Co1	0.5000	0.0000	1.0000	0.01887 (13)	
C1	0.1280 (2)	0.2122 (2)	1.01741 (17)	0.0240 (4)	
C2	−0.0189 (2)	0.2764 (2)	0.93891 (16)	0.0236 (4)	
H2A	−0.1265	0.2227	0.9683	0.028*	
H2B	−0.0468	0.3982	0.9496	0.028*	
C3	0.0277 (3)	0.0817 (2)	0.76392 (18)	0.0262 (4)	
H3A	−0.0235	0.0016	0.8182	0.031*	
C4	0.0928 (3)	0.0397 (2)	0.64190 (19)	0.0305 (4)	
H4A	0.0861	−0.0691	0.6131	0.037*	
C5	0.1687 (3)	0.1600 (2)	0.56150 (18)	0.0280 (4)	
H5A	0.2178	0.1303	0.4797	0.034*	
C6	0.1715 (2)	0.3251 (2)	0.60302 (17)	0.0218 (4)	
C7	0.1054 (2)	0.3596 (2)	0.72797 (17)	0.0219 (4)	
H7A	0.1075	0.4681	0.7590	0.026*	
C8	0.2383 (2)	0.4612 (2)	0.51779 (16)	0.0217 (4)	
C9	0.2396 (2)	0.4480 (2)	0.38854 (17)	0.0243 (4)	
H9A	0.1995	0.3532	0.3564	0.029*	
C10	0.3003 (2)	0.5750 (2)	0.30783 (17)	0.0237 (4)	
H10A	0.3012	0.5640	0.2219	0.028*	
C11	0.3597 (2)	0.7182 (2)	0.35321 (16)	0.0221 (4)	
C12	0.3616 (2)	0.7305 (2)	0.48192 (17)	0.0235 (4)	
H12A	0.4038	0.8246	0.5135	0.028*	
C13	0.3014 (2)	0.6043 (2)	0.56340 (16)	0.0239 (4)	
H13A	0.3028	0.6148	0.6491	0.029*	
C14	0.4175 (2)	0.8612 (2)	0.26645 (17)	0.0225 (4)	
O1	0.23496 (18)	0.08802 (17)	0.97274 (13)	0.0280 (3)	
O2	0.1218 (2)	0.2815 (2)	1.12000 (14)	0.0381 (4)	
O3	0.4574 (2)	0.99102 (19)	0.31444 (13)	0.0371 (4)	
O4	0.41823 (18)	0.83772 (17)	0.14971 (12)	0.0268 (3)	
O5	0.5063 (2)	−0.20597 (18)	0.87341 (14)	0.0309 (3)	
H5C	0.629 (4)	−0.250 (4)	0.867 (3)	0.046*	
H5B	0.504 (4)	−0.150 (4)	0.806 (3)	0.046*	
O6	0.3383 (3)	0.4835 (3)	−0.0904 (2)	0.0637 (6)	
H6A	0.437 (4)	0.402 (3)	−0.100 (4)	0.096*	
H6B	0.399 (5)	0.578 (3)	−0.108 (4)	0.096*	
N1	0.03825 (19)	0.23849 (19)	0.80482 (14)	0.0213 (3)	

### Atomic displacement parameters (Å^2^)

**Table:**

	*U*^11^	*U*^22^	*U*^33^	*U*^12^	*U*^13^	*U*^23^
Co1	0.02626 (19)	0.01584 (18)	0.01403 (18)	−0.00255 (12)	−0.00149 (12)	0.00310 (12)
C1	0.0299 (9)	0.0220 (9)	0.0202 (9)	−0.0061 (7)	−0.0021 (7)	0.0069 (7)
C2	0.0257 (8)	0.0247 (9)	0.0185 (8)	−0.0008 (7)	0.0008 (7)	0.0034 (7)
C3	0.0321 (9)	0.0213 (9)	0.0253 (9)	−0.0061 (7)	−0.0028 (7)	0.0064 (7)
C4	0.0403 (11)	0.0208 (9)	0.0299 (10)	−0.0037 (8)	−0.0029 (8)	0.0005 (8)
C5	0.0342 (10)	0.0267 (9)	0.0212 (9)	−0.0012 (8)	0.0002 (7)	0.0008 (7)
C6	0.0225 (8)	0.0227 (9)	0.0196 (8)	−0.0007 (7)	−0.0023 (6)	0.0046 (7)
C7	0.0252 (8)	0.0193 (8)	0.0212 (9)	−0.0030 (7)	−0.0037 (7)	0.0048 (7)
C8	0.0217 (8)	0.0227 (9)	0.0189 (9)	0.0004 (6)	−0.0003 (6)	0.0056 (7)
C9	0.0276 (9)	0.0244 (9)	0.0211 (9)	−0.0038 (7)	−0.0032 (7)	0.0028 (7)
C10	0.0255 (8)	0.0283 (9)	0.0164 (8)	−0.0003 (7)	−0.0022 (6)	0.0049 (7)
C11	0.0212 (8)	0.0241 (9)	0.0191 (9)	0.0012 (7)	0.0003 (6)	0.0066 (7)
C12	0.0277 (9)	0.0220 (9)	0.0202 (9)	−0.0018 (7)	−0.0021 (7)	0.0025 (7)
C13	0.0292 (9)	0.0256 (9)	0.0156 (8)	−0.0011 (7)	−0.0011 (7)	0.0028 (7)
C14	0.0250 (8)	0.0226 (9)	0.0183 (8)	0.0007 (7)	−0.0003 (6)	0.0043 (7)
O1	0.0301 (7)	0.0252 (7)	0.0285 (7)	0.0012 (5)	−0.0071 (5)	0.0014 (5)
O2	0.0499 (9)	0.0381 (8)	0.0265 (7)	0.0040 (7)	−0.0121 (6)	−0.0041 (6)
O3	0.0628 (10)	0.0288 (7)	0.0219 (7)	−0.0151 (7)	−0.0066 (7)	0.0050 (6)
O4	0.0387 (7)	0.0241 (6)	0.0173 (6)	−0.0066 (5)	−0.0011 (5)	0.0055 (5)
O5	0.0476 (9)	0.0226 (7)	0.0229 (7)	−0.0053 (6)	−0.0060 (6)	0.0021 (5)
O6	0.0462 (10)	0.0515 (11)	0.0891 (16)	−0.0104 (9)	0.0100 (10)	−0.0085 (11)
N1	0.0232 (7)	0.0222 (7)	0.0179 (7)	−0.0008 (6)	−0.0031 (6)	0.0050 (6)

### Geometric parameters (Å, º)

**Table:**

Co1—O4^i^	2.1031 (12)	C7—N1	1.346 (2)
Co1—O4^ii^	2.1031 (12)	C7—H7A	0.9300
Co1—O1	2.1184 (13)	C8—C9	1.396 (3)
Co1—O1^iii^	2.1184 (13)	C8—C13	1.400 (3)
Co1—O5^iii^	2.1392 (14)	C9—C10	1.387 (3)
Co1—O5	2.1392 (14)	C9—H9A	0.9300
C1—O2	1.236 (2)	C10—C11	1.387 (3)
C1—O1	1.263 (2)	C10—H10A	0.9300
C1—C2	1.533 (3)	C11—C12	1.394 (2)
C2—N1	1.474 (2)	C11—C14	1.512 (2)
C2—H2A	0.9700	C12—C13	1.385 (3)
C2—H2B	0.9700	C12—H12A	0.9300
C3—N1	1.340 (2)	C13—H13A	0.9300
C3—C4	1.370 (3)	C14—O3	1.244 (2)
C3—H3A	0.9300	C14—O4	1.274 (2)
C4—C5	1.387 (3)	O4—Co1^iv^	2.1031 (12)
C4—H4A	0.9300	O5—H5C	0.95 (3)
C5—C6	1.393 (3)	O5—H5B	0.85 (3)
C5—H5A	0.9300	O6—H6A	0.927 (18)
C6—C7	1.389 (3)	O6—H6B	0.924 (18)
C6—C8	1.483 (2)		
			
O4^i^—Co1—O4^ii^	180.0	C5—C6—C8	122.02 (17)
O4^i^—Co1—O1	86.13 (5)	N1—C7—C6	120.95 (16)
O4^ii^—Co1—O1	93.87 (5)	N1—C7—H7A	119.5
O4^i^—Co1—O1^iii^	93.87 (5)	C6—C7—H7A	119.5
O4^ii^—Co1—O1^iii^	86.13 (5)	C9—C8—C13	118.49 (16)
O1—Co1—O1^iii^	180.000 (1)	C9—C8—C6	119.86 (16)
O4^i^—Co1—O5^iii^	88.97 (5)	C13—C8—C6	121.65 (16)
O4^ii^—Co1—O5^iii^	91.03 (5)	C10—C9—C8	120.49 (17)
O1—Co1—O5^iii^	88.79 (6)	C10—C9—H9A	119.8
O1^iii^—Co1—O5^iii^	91.21 (6)	C8—C9—H9A	119.8
O4^i^—Co1—O5	91.03 (5)	C11—C10—C9	121.02 (16)
O4^ii^—Co1—O5	88.97 (5)	C11—C10—H10A	119.5
O1—Co1—O5	91.21 (6)	C9—C10—H10A	119.5
O1^iii^—Co1—O5	88.79 (6)	C10—C11—C12	118.66 (16)
O5^iii^—Co1—O5	180.0	C10—C11—C14	121.32 (16)
O2—C1—O1	127.55 (18)	C12—C11—C14	120.01 (17)
O2—C1—C2	116.33 (17)	C13—C12—C11	120.78 (17)
O1—C1—C2	115.98 (16)	C13—C12—H12A	119.6
N1—C2—C1	111.06 (14)	C11—C12—H12A	119.6
N1—C2—H2A	109.4	C12—C13—C8	120.54 (16)
C1—C2—H2A	109.4	C12—C13—H13A	119.7
N1—C2—H2B	109.4	C8—C13—H13A	119.7
C1—C2—H2B	109.4	O3—C14—O4	125.88 (17)
H2A—C2—H2B	108.0	O3—C14—C11	117.89 (16)
N1—C3—C4	119.77 (17)	O4—C14—C11	116.22 (16)
N1—C3—H3A	120.1	C1—O1—Co1	132.41 (12)
C4—C3—H3A	120.1	C14—O4—Co1^iv^	127.67 (12)
C3—C4—C5	119.77 (18)	Co1—O5—H5C	100.6 (17)
C3—C4—H4A	120.1	Co1—O5—H5B	99.2 (19)
C5—C4—H4A	120.1	H5C—O5—H5B	102 (2)
C4—C5—C6	120.13 (18)	H6A—O6—H6B	98 (2)
C4—C5—H5A	119.9	C3—N1—C7	121.83 (16)
C6—C5—H5A	119.9	C3—N1—C2	118.73 (15)
C7—C6—C5	117.43 (16)	C7—N1—C2	119.39 (15)
C7—C6—C8	120.53 (16)		

Symmetry codes: (i) −*x*+1, −*y*+1, −*z*+1; (ii) *x*, *y*−1, *z*+1; (iii) −*x*+1, −*y*, −*z*+2; (iv) *x*, *y*+1, *z*−1.

### Hydrogen-bond geometry (Å, º)

**Table:**

*D*—H···*A*	*D*—H	H···*A*	*D*···*A*	*D*—H···*A*
O5—H5*C*···O2^iii^	0.95 (3)	1.91 (3)	2.835 (2)	164 (3)
O5—H5*B*···O3^i^	0.85 (3)	1.80 (3)	2.617 (2)	162 (3)
O6—H6*A*···O4^v^	0.93 (2)	2.13 (2)	3.003 (2)	156 (3)
O6—H6*B*···O5^iv^	0.92 (2)	1.96 (2)	2.880 (2)	173 (4)

Symmetry codes: (i) −*x*+1, −*y*+1, −*z*+1; (iii) −*x*+1, −*y*, −*z*+2; (iv) *x*, *y*+1, *z*−1; (v) −*x*+1, −*y*+1, −*z*.
